# Cost containment analysis of superparamagnetic iron oxide (SPIO) injection in patients with ductal carcinoma in situ

**DOI:** 10.1007/s10549-024-07451-2

**Published:** 2024-08-01

**Authors:** Odette Solís, Jamin Addae, Raeshell Sweeting, Ingrid Meszoely, Ana Grau, Rondi Kauffmann, Mark Kelley, Rachel McCaffrey, Kelly Hewitt

**Affiliations:** Division of Surgical Oncology & Endocrine Surgery, Vanderbilt University Medical Center, Preston Research Building, 597, 2220 Pierce Avenue, Nashville, TN 37232 USA

**Keywords:** Ductal carcinoma in situ, Mastectomy, SPIO, Cost analysis

## Abstract

**Purpose:**

Recent studies have established the safety and efficacy of Superparamagnetic Iron Oxide (SPIO, Magtrace®) for delayed sentinel lymph node biopsy (SLNB) in patients with ductal carcinoma in situ (DCIS) who are undergoing mastectomy. The aim of our study was to measure cost containment with use of Magtrace® in comparison to upfront SLNB with traditional technetium-99 lymphatic tracer.

**Methods:**

A total of 41 patients at our institution underwent mastectomy with Magtrace® injection for DCIS and were included in our single-institution, retrospective analysis. For comparison, total charges data were obtained for an upfront SLNB at the time of mastectomy. Cost comparison analysis was then performed against charges for intraoperative Magtrace® injection with additional charges incorporated for those patients who required return to the operating room for delayed SLNB. Total cost containment for the cohort with use of Magtrace® was then measured.

**Results:**

Of the 41 patients who underwent Magtrace® injection, two patients required return to the operating room for a delayed SLNB for invasive disease. Including these charges for a second encounter into our cost analysis, the use of Magtrace® still yielded an overall cost containment of $205,793.55 in our cohort when comparing to patients who underwent upfront SLNB. For patients who underwent Magtrace® injection and did not require return to the operating room, charges were reduced by $6,768.52 per patient.

**Conclusion:**

The use of Magtrace® for delayed SLNB in patients with DCIS undergoing mastectomy yielded a significant overall cost containment, further supporting its use in this patient population.

## Introduction

As screening and detection of breast cancer continues to improve, there has been a concomitant increase in detection of ductal carcinoma in situ (DCIS), or preinvasive breast cancer [1]. Surgical options for patients with a diagnosis of DCIS include breast conservation or mastectomy. The incidence of nodal positivity in patients with pure DCIS on final pathology is low, and in some studies cited to be less than 1% [2]. In the setting of breast conserving surgery, sentinel lymph node biopsy is usually not recommended unless final pathology reveals invasive disease. By contrast, patients with DCIS undergoing mastectomy often undergo sentinel lymph node biopsy at the time of mastectomy, as complete mastectomy disrupts lymphatic channels and precludes future axillary mapping if invasive disease is found on final pathology. Current NCCN guidelines therefore strongly recommend that patients undergoing mastectomy for DCIS also undergo a sentinel lymph node biopsy at the time of surgery to obtain axillary staging in case final pathology demonstrates invasive disease [3]. The rate of upstage from DCIS on core biopsy to invasive cancer on final pathology is variable and reported between 9 and 44% in the current literature [4–7].

Superparamagnetic iron oxide (SPIO, Magtrace®) obtained FDA approval in 2018 as an alternative lymphatic tracer. Unlike standard dual tracers of radioactive technetium (Tc-99) and blue dye, SPIO has the advantage of retention in the lymphatics for at least 30 days following injection [8]. This advantage has been utilized in patients with DCIS undergoing mastectomy to avoid an upfront sentinel lymph node biopsy and allow the option of delayed sentinel lymph node biopsy for the small subset of patients who are found to have invasive carcinoma on final pathology.

The initial SentiNot trial established the effectiveness of Magtrace for delayed sentinel lymph node biopsy, in addition to cost containment [9]. However, these studies were conducted primarily in the Swedish healthcare system and there remains a question of whether that cost savings would translate to the healthcare system in the United States. The aim of our study was to perform a cost containment analysis within our single, US-based institution.

## Methods

Institutional IRB approval was obtained prior to data collection. Patients undergoing mastectomy for DCIS with use of Magtrace® for delayed sentinel node detection between August 2021 and January 2023 were collected for our analysis. Regarding injection technique, 2 cc of Magtrace® were injected into the deep subareolar space at the time of mastectomy after induction of anesthesia but prior to pectoral block procedure. For patients undergoing Tc-99 injection in our comparison group, it is our institution’s practice to have this performed pre-operatively on the day of surgery in the Nuclear Medicine department. Various mastectomy types were then performed. Total charges data were obtained from our single institution for these 41 patients, including charges associated with the additional encounter for the two patients who required return to the operating room for a delayed sentinel lymph node biopsy.

For our comparison, total charges data for an upfront sentinel lymph node biopsy at the time of mastectomy were also obtained. This included charges for additional operative and anesthesia time, charges associated with lymphatic tracer injection, and pathology and specimen processing charges. For a sentinel lymph node biopsy at the time of initial mastectomy, we estimated it would take an average of 30 min additional operating room time. With regard to lymphatic tracer, charges for Tc-99 injection were included in our comparison group but isosulfan blue was excluded from our analysis because not all surgeons in the study group utilized blue dye for dual tracer. Pathology charges for additional specimen processing and report were included for our comparison group as well.

Once total charges data were gathered, a charges comparison of the Magtrace® cohort and upfront sentinel node cohort was completed. We calculated the overall cost containment for this cohort, per patient cost reduction for those patients undergoing Magtrace® injection who did not require a return to the operating room, and additional charges associated with those two patients who did require return to the operating room for delayed sentinel lymph node biopsy. Based on our charges data, we then calculated the rate of return to the operating room at which Magtrace® use for delayed sentinel node would no longer be cost effective.

## Results

Patient demographic data for the Magtrace® cohort are included in Table 1, including race and ethnicity distribution. The most common indication for mastectomy in our DCIS cohort was diffuse disease (31.7%). The most common type of mastectomy performed was skin-sparing (48.9%). Of the 41 patients who received Magtrace® (45 mastectomies), two patients required return to the operating room for delayed sentinel lymph node biopsy, with rate of return to the operating room at 5%. The timing to return to the operating room was 27 days and 18 days for each patient. An average of 3 lymph nodes were removed for both the upfront sentinel lymph node biopsy cohort, as well as for the two patients who required return to the operating room. Charges for each of these additional encounters for return to the operating room were $35,041.54 and $36,674.23, respectively.

**Table 1:** Patient demographics

Total cohort	*N* = 41
Age, median	58 (IQR = 17, range 25–76)
Sex (%)	
Female	97.6
Race (%)	
White	82.9
Black	12.2
Other	4.9
Body mass index (BMI)	27.7 (IQR = 6.9)
Insurance (%)	
Medicaid	2.4
Medicare	22
Private	75.6
Indication for mastectomy (%)	
Diffuse disease	31.7
Patient choice	26.8
High risk	19.5
Multifocal disease	12.2
Close to nipple	4.9
Previous radiation	2.4
Unknown	2.4
Mastectomies	*N* = 45
Type of mastectomy	
Skin-sparing	48.9
Total	28.9
Nipple-sparing	22.2
Reconstruction	
Yes	71.1
No	28.9

Table 2 illustrates charges data associated with upfront sentinel lymph node biopsy in comparison to our Magtrace® cohort. At our institution, the pathology charge for sentinel lymph nodes is a standard fixed charge regardless of number of nodes removed. Our cost containment calculation measured the difference between total charges for an upfront sentinel lymph node biopsy at $359,591.32 (bolded, Table 2) and the charges for Magtrace(R) and charges associated with the two returns to the operating room at $153,797.77 (bolded, Table 2), yielding an overall cost containment of $205,793.55. Even considering additional charges associated with the two patients who required return to the operating room, Magtrace® still yielded a significant overall cost containment compared to upfront sentinel lymph node biopsy for all DCIS patients undergoing mastectomy.

**Table 2:** Cost containment analysis

Cost containment =	Description	#Patients	Charges/patient	Total charges
Charges of upfront sentinel lymph node biopsy (SLNB)	Additional 30 + min operating room time	41	$892.00	$36,572.00
	Additional 30 + min anesthesia time	41	$3,135.00	$128,535.00
	Technetium-99 (Tc-99) charge	41	$1,974.52	$80,955.32
	Tc-99 injection procedure	41	$782.00	$32,062.00
	SLNB pathology	41	$425.00	$17,425.00
	SLNB procedure	41	$1,562.00	$64,042.00
				**$359,591.32**
–				
Charges of magtrace + 2 returns to the operating room (RTOR)	Magtrace®	41	$2,002.00	$82,082.00
	RTOR encounter charges	1	$35,041.54	$35,041.54
		1	$36,674.23	$36,674.23
				**$153,797.77**
=				
Total cost containment				**$205,793.55**

For a patient who underwent Magtrace® injection with pure DCIS on final pathology who did not require return to the operating room for delayed sentinel lymph node biopsy, cost reduction was estimated at $6,768.52 per patient in comparison to charges for an upfront sentinel lymph node biopsy. This accounted for 39 of 41 of patients in this cohort, or 95% of patients. For the two patients (remaining 5%) who required a return to the operating room, net additional cost was estimated at $27,087.37 per patient on average. Both of these patients successfully mapped and an average of 3 lymph nodes were removed on return to the operating room. Taking these charges into account led to an estimated cost containment of $205,793.55 for the entire cohort. As mentioned, our rate of return to the operating room was 5% (2 patients). We estimated that in order for Magtrace® to no longer be cost effective in this cohort, our rate of return to the operating room would need to exceed 17%.

## Discussion

Surgical risks associated with sentinel lymph node biopsy have been well documented and are not insignificant, including risks of lymphedema and wound complications [10]. Although current guidelines recommend upfront sentinel lymph node biopsy at the time of mastectomy for DCIS, several studies have now established the feasibility and safety of Magtrace® injection in an effort to de-escalate axillary surgery in this patient population [8, 11]. The purpose of our study was to further investigate the impact of de-escalation on healthcare costs by performing a cost containment analysis. For many clinicians, a common barrier to adopting new technology into practice is cost to their healthcare institution. To our knowledge, this is the first study to assess cost in this patient population within a single US-based healthcare system.

Our study not only included charges for each lymphatic tracer in its comparison, but also included the less obvious charges associated with an upfront sentinel lymph node biopsy. This includes additional operating room and anesthesia charges in order to perform a sentinel lymph node biopsy at the time of mastectomy, as well as charges related to pathology processing and analysis. Our study did not include charges for isosulfan blue at the time of mastectomy, as not all surgeons in our cohort used dual tracer, but this in theory would have further increased cost containment in favor of Magtrace® use.

It is important to note that charges vary by institution and there is significant variability by institution regarding technetium injection that influence charges. Our institution’s practice is to have patients taken from the preoperative holding area down to the Nuclear Medicine department for technetium injection on the day of surgery. These charges were included in our analysis, but there may be significant variability in charges compared to an institution where, for example, surgeons are injecting technetium in the operating room prior to incision. In addition, our analysis did not consider upfront costs associated with purchasing the reusable Sentimag® probe, which our institution already owned and utilized for Magseed® localization. Within a system that does not already own this technology for Magseed® detection, this would be an additional upfront institutional cost that would need to be factored into a cost containment analysis.

Previous studies have established the safety and feasibility of using Magtrace® to de-escalate axillary surgery in patients with DCIS undergoing mastectomy. Our study further encourages this shift in practice with the additional benefit of cost containment, offering practical and relevant data when considering management options for patients with DCIS.

## Data Availability

No datasets were generated or analysed during the current study.
